# The First Symposium on Organ Donation in Collaboration with Spain

**Published:** 2011-11-01

**Authors:** M. R. Masjedi, K. Najafizadeh, S. Shafaghi

**Affiliations:** 1 *Symposium President, *; 2 *Symposium Scientific Director,*; 3 *Symposium Executive Director*

## INTRODUCTION

the Organ Procurement and Transplant Unit of Shahid Beheshti University of Medical Sciences (SBMU-OPTU) in collaboration with the Transplant Procurement Management of Spain Group (TPM) had the pleasure to organize “the 1^st^ symposium on organ donation in collaboration with Spain” in July 25–27, 2011 which was held in Tehran, the capital city of Iran.

Considering Iranian patients’ growing need for organs donated by brain-dead patients and the low rate of organ donation in Iran compared to that in other countries, it is quite necessary to eliminate the existing obstacles to organ donation and improve the efficiency of organizations dealing with organs procurement.

In 2010, there were only some 4.3 organ donors per one million population in Iran while, on average, the rate was 10–20 donors per one million population in other countries. In Spain which is considered a leading country in terms of organ donation, there were some 35 organ donors per one million population in 2010. 

With regard to the success stories of Spain and the country’s long-held experience in educating volunteers about different stages of organ donation, SBMU-OPTU decided to invite Spanish organ donation experts to train donor coordinators and main personnel of organ donation organizations in Iran. 

The training course consisted of three one-day workshops as follows:

- Donor Selection Criteria
- Family Approach for Organ Donation and Brain Death Management
- Organ Allocation Criteria and Tissue Banking Organization

## PARTICIPANTS

The target audience of the training course was the organ procurement officials including physicians and nurses (including anesthesiologists, neurologists, neurosurgeons, and general practitioners) who deal with patients affected by brain death as well as social workers who deal with getting consent from the family members of the brain-dead potential donors. 

## WORKSHOPS

For convenience, the course was held on three consecutive days. The sessions, both theoretical and practical, were held from 8:00 am to 5:00 pm with some breaks.

## TRANSLATION FACILITIES

All syllabi presented by the Spanish team were translated into Persian by an interpreter. All presentations and speeches in Persian as well as questions raised by Iranian audience were also translated simultaneously into English.

## ACHIEVEMENTS OF THE SYMPOSIUM

Coordinators working in Tehran and other provinces were given the opportunity for exchanging information and experience.

The TPM team from Brazil and Spain were made aware of developments, techniques, tireless efforts and achievements of transplant teams in Iran.

A meeting is supposed to be held to develop a protocol for organ donation in the Ministry of Health.

Before the Symposium, all transplant teams in Iran only preferred to use organs of ideal donors. After the Symposium, more attention was paid to the marginal donors including the old donors. The brilliant result of this change was the first-ever 66-year-old donor whose kidneys were transferred to Labafi Nejad and Baqiatallah Hospitals without any problem. Fortunately, both kidneys and old recipients are doing well.

Authorities of the Ministry of Health were informed about the challenges of Transplant Teams through the information provided by organ procurement units of Tehran and other provinces.

Strong motivation was provided for the Transplant Team of Shahid Beheshti University of Medical Sciences and other universities to continue their efforts more effective than ever. They decided to have a fundamental change in their operation and found out that they have more potential ability to increase the number of their brain-dead donors.

All three days of the Symposium were recorded in order to be used in future for training purposes.

Data bank of coordinators and heads of organ donation teams was compiled and given to all units of organ donation and procurement.

Finally, all teams and participants expressed their great satisfaction and appreciation about this influential Symposium.

## SPECIAL ASPECTS (FEATURES) OF THE SSYMPOSIUM:

Of the unique features of the Symposium, mentioned by the Spanish team, was the possibility of the effective simultaneous translation which was not only an eye-catching positive aspect of this event, but also played a key role in easy and clear communication between both sides.

The Symposium was held in an appropriate place, equipped with high-tech facilities and services which had a significant effect on the quality of the whole program.

Training of case selection was done by a computer animation.

Since two film-makers from the Spain TV accompanied the team, the good impression of the team about organ donation and transplantation in Iran will be transferred to the world.

You can also find Dr. Manialich’s letter as follows, which contains his views about this symposium, the process of organ donation in Iran and future plans as well as continuous collaboration and cooperation between Iran and Spain.

*Dear Colleagues* *,*

Thanks a lot for your hospitality and support to my colleagues, and the film-maker, during all the time in Iran.

I will try to summarize our impression for the visit, our future plans and strategy for near future, and answer your last e-mail.

Visit impressions

Well motivate team, well established and running OPU’s with potential donors, recipient and transplanted patients, and hospital professional support.

Nice organization of the Symposium, well attended from all Iran and fully supported by experts and recognized professionals, Ministry of Health and pharmaceutical companies.

Future Plan Strategy

I have been discussing with TPM (Gloria Paez) and DTI (Maria Paula Gomez) our conditions for the future thought an agreement between OPU and University and TPM-DTI Foundation to develop three points:

1. To improve your OPUs in Iran, implementing SEUSA Programme: a) increasing referrals DAS and audit; b) increasing conversion rate; and c) improving structure.
2. To help Ministry of Health in developing a sharing office and registry for organ allocation.
3. To develop a National TPM Advanced Course for the next 3-5 years together with you for all Iran.

